# Study of Industrial Grade Thermal Insulation at Elevated Temperatures

**DOI:** 10.3390/ma13204613

**Published:** 2020-10-16

**Authors:** Amalie Gunnarshaug, Maria Monika Metallinou, Torgrim Log

**Affiliations:** 1 Q Rådgivning AS, 5527 Haugesund, Norway; amg@q-rad.no; 2 Department of Physics and Technology, University of Bergen, 5020 Bergen, Norway; 3 Fire Disasters Research Group, Department of Safety, Chemistry and Biomedical Laboratory Sciences, Western Norway University of Applied Sciences, 5528 Haugesund, Norway; torgrim.log@hvl.no

**Keywords:** industrial thermal insulation, passive fire protection, hydrocarbon fires, thermal conductivity, TGA, DSC, TPS

## Abstract

Thermal insulation is used for preventing heat losses or heat gains in various applications. In industries that process combustible products, inorganic-materials-based thermal insulation may, if proven sufficiently heat resistant, also provide heat protection in fire incidents. The present study investigated the performance and breakdown temperature of industrial thermal insulation exposed to temperatures up to 1200 °C, i.e., temperatures associated with severe hydrocarbon fires. The thermal insulation properties were investigated using thermogravimetric analysis (TGA), differential scanning calorimetry (DSC) and by heating 50 mm cubes in a muffle furnace to temperatures in the range of 600 to 1200 °C with a 30 min holding time. The room temperature thermal conductivity was also recorded after each heat treatment. Upon heating, the mineral-based oil dust binder was released at temperatures in the range of 300 to 500 °C, while the Bakelite binder was released at temperatures in the range of 850 to 960 °C. The 50 mm test cubes experienced increasing levels of sintering in the temperature range of 700 to 1100 °C. At temperatures above 1100 °C, the thermal insulation started degrading significantly. Due to being heat-treated to 1200 °C, the test specimen morphology was similar to a slightly porous rock and the original density of 140 kg/m^3^ increased to 1700 kg/m^3^. Similarly, the room temperature thermal conductivity increased from 0.041 to 0.22 W/m∙K. The DSC analysis confirmed an endothermic peak at about 1200 °C, indicating melting, which explained the increase in density and thermal conductivity. Recently, 350 kW/m^2^ has been set as a test target heat flux, i.e., corresponding to an adiabatic temperature of 1200 °C. If a thin layer of thermally robust insulation is placed at the heat-exposed side, the studied thermal insulation may provide significant passive fire protection, even when exposed to heat fluxes up to 350 kW/m^2^. It is suggested that this is further analysed in future studies.

## 1. Introduction

Thermal insulation is widely used in several application areas, such as in the building industry, refrigeration plants and in the process industries. In the process industries, examples of typical application areas may be temperature control, personnel protection, humidity condensation prevention or sound attenuation. In the hydrocarbon process industry, thermal insulation may be necessary in order to maintain the required production temperature [1]. A typical example may be distillation columns; in order to obtain good production efficiency and quality of the distilled products, the temperature profiles are carefully designed. These types of process equipment may represent a potential for a major accidental hazard, as it may release large quantities of flammable material upon a potential leak. Ignition of a hydrocarbon leak may lead to severe heat loads to the exposed object, i.e., flame temperatures in the range of 1100 to 1200 °C, corresponding to heat loads in the range of 250 to 350 kW/m^2^ [2,3]. These high heat fluxes will result in a significant temperature increase in exposed production units, which may result in weakening of the steel and a possible escalation of the fire scenario, especially if the steel temperature exceeds 500 °C [4]. The outcome of a process equipment rupture may be a disastrous release, as evidenced by several major accidents during the last decade [5,6,7]. Hence, these types of units are also often protected with an additional layer of mineral-based passive fire protection.

The fire resistance of the passive fire protection is normally given as a time in minutes until an object reaches a specified critical temperature. In the oil and gas industry, the critical steel temperature is conservatively set to 400 °C, i.e., the protected element should not achieve temperatures above 400 °C within the specified time frame [8]. The fire resistance is typically given in intervals of 15, 30, 60 or 120 min [9].

**Figure 1:**
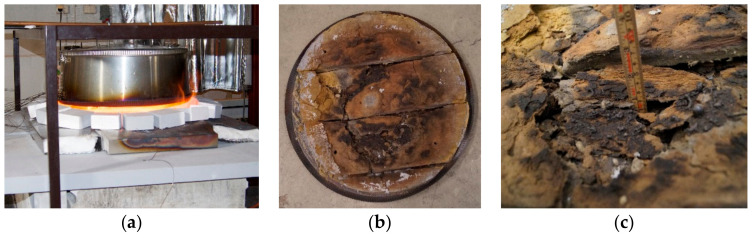
Small-scale jet-fire test setup (**a**), exposed thermal insulation after the jet fire testing (**b**) and melted and sintered remains after high heat flux (350 kW/m^2^) fire testing (**c**) [10,11].

Previous studies of 50 mm thick thermal insulation (ProRox PSM 971, 50 mm, Rockwool) protecting a 16 mm thick steel wall using small scale testing [10,11] rather than full-scale testing [12] demonstrated that the thermal insulation alone was sufficient to withstand 30 min of jet fire exposure. Even with only 3 mm thick steel walls, the testing showed sufficient fire protection for 20 min. During the testing, the thermal insulation sintered and partially melted in some locations. The sintering and melting of the insulation due to the heat exposure resulted in cracks/openings in the insulation mat, as shown in Figure 1b,c. The previous oven testing up to 1100 °C [11] showed minimal shrinking (less than 25%) of the thermal insulation. Hence, a further examination of the thermal insulation, explaining the observations in the small-scale jet fire testing [10,11] and determining the breakdown temperature of the insulation are the motivations for the present study.

Sjöström et al. [13] and Olsen et al. [14], performed oven tests of a Rockwool insulation similar to that of Bjørge et al. [11], i.e., recording the temperature in the centre of the insulation. In addition, thermogravimetric analysis (TGA)/differential scanning calorimetry (DSC) tests and transient plane source method (TPS) measurements were performed. However, the scope of their studies [13,14,15,16,17] was limited to temperatures associated with building fires, i.e., at temperatures up to 1000 °C. Their studies focused on the performance of stone wool in such fires [13,14,16] and an analysis of the properties (measured using TGA/DSC/TPS) within the operating range of that thermal insulation [15,17].

Several numerical models have been developed that calculate the fire resistance of insulated walls or columns [18,19,20]. In order to account for the breakdown of the thermal insulation during fire exposure, the conductivity has, e.g., been adjusted in order to make the model fit with the performed fire tests. This has in some cases overestimated or underestimated the actual conductivity and breakdown of the insulation. The properties of the thermal insulation (Rockwool) at temperatures above the normal operating temperatures are generally missing in the literature.

The present study aimed at investigating the properties of the thermal insulation after being exposed to temperatures up to the breakdown temperature of the insulation. Cubes of the thermal insulation (50 mm) were heat-treated in a muffle furnace at different exposure temperatures up to 1200 °C. To support the findings from the muffle oven tests and further investigate the properties of the thermal insulation, in-depth analyses of the material were performed. To reveal the mass loss at elevated temperatures, thermogravimetric analysis (TGA) was performed at temperatures up to 1250 °C. To examine the melting temperature of the thermal insulation, DSC to 1250 °C was performed. The ambient temperature thermal conductivity of test specimens preheated up to 1200 °C was measured using TPS.

The materials and methods used are explained in Section 2. Section 3 presents the results from the furnace tests, the TGA and DSC analyses and the results from the TPS measurements. Section 4 presents the discussions and Section 5 presents the overall conclusions and suggestions for future studies.

## 2. Materials and Methods

### 2.1. The Studied Thermal Insulation

In the present study, industrial-grade pipe section mat (ProRox PSM 971, thickness 50 mm, Trondheim, Norway) delivered by Rockwool, Inc., was studied as a representative industrial thermal insulation, i.e., the same thermal insulation as in previous studies [10,11]. The detailed technical data and thermal conductivity of this thermal insulation up to 350 °C are presented in Appendix A, Table A1 and Table A2. The maximum service temperature of the studied insulation, as given by the manufacturer, is 700 °C, which is well below temperatures associated with fires in the oil and gas industry. Temperatures above the service temperature were therefore focused on in the present study.

Chemically, the main components of the thermal insulation are inorganic oxides. The thermal insulation mainly consists of silica, alumina, magnesia, calcium oxide and iron (III) oxide. In addition, there are minor amounts of sodium oxide, potassium oxide, titanium oxide and phosphorous pentoxide. The detailed chemical composition, as received from the supplier, is presented in Appendix A, Table A3.

The thermal insulation is produced by melting the raw materials at 1500 °C before it is cooled and spun into insulation mats [21]. In addition, a dust binder is added (mineral-based oil) to make the material easier to handle when, e.g., cutting and fitting the insulation mat to equipment requiring thermal insulation. Bakelite, i.e., polyoxybenzylmethylenglycolanhydride (C_6_H_6_O ∙ CH_2_O)_x_, is also added to give some strength to the thermal insulation up to the maximum service temperature.

As the insulation is heated, the mineral oil will gradually pyrolyse/evaporate. The degradation process of the Bakelite is dependent on the actual production conditions and the degradation process may be complicated [22]. The number of molecular cross-links will influence the degradation processes and there may be several reaction paths. Generally, the degradation of Bakelite may be expressed as the following non-balanced reaction:

(C_6_H_6_O ∙ CH_2_O)_x_ → CO_2_ + CO + H_2_O + C_soot_ + other products. (1)

The number of cross-links, in addition to other components mixed into the Bakelite, will have an impact on the degradation temperatures [22].

### 2.2. Thermal Conductivity

**Figure 2:**
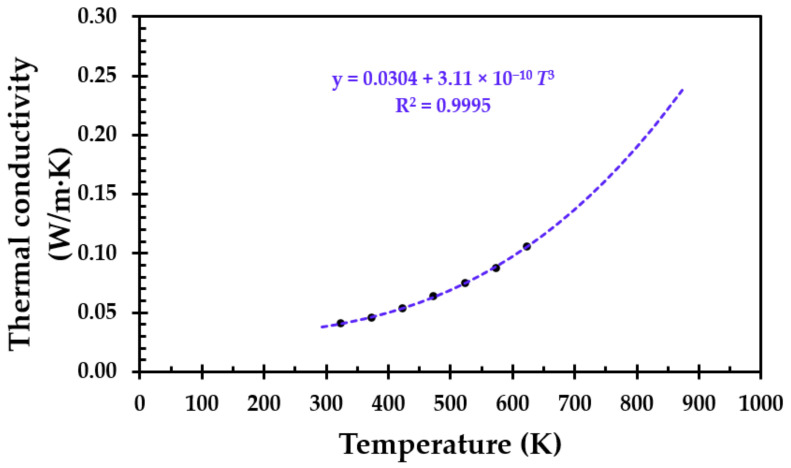
Thermal conductivity of the thermal insulation (ProRox PSM 971, 50 mm) as a function of the absolute temperature. Data from Appendix A, Table A2.

For materials like, e.g., thermal insulation, the thermal conductivity is limited by the pore radiation. Theoretically, it can be shown that the thermal conductivity (*k*) will be proportional to the absolute temperature to the third power, i.e., *T*^3^ [23]. The thermal conductivity of the virgin thermal insulation as a function of absolute temperature is presented in Figure 2. It can be very well described by Equation (2), which is also presented in Figure 2.

*k* = 0.0304 + 3.11 × 10^−10^*T*^3^. (2)

The good fit of Equation (2), i.e., R^2^ = 0.9995, with a major contribution of the temperature dependency to the third power, i.e., *T*^3^, clearly indicates that the thermal conductivity for this particular thermal insulation is indeed limited by pore radiation. However, when the breakdown of the thermal insulation is significant, the thermal conductivity may no longer be limited by pore radiation and is thus assumed to increase.

When exposing inorganic (ceramic) materials to elevated temperatures, sintering of the material may occur, i.e., an entropy-driven [24] physical process leading to a lower free energy, ΔG. To limit sharp edges and optimise the mix of the material, the atoms in contacting threads will diffuse across the thread boundaries. With time, theoretically, the material may approach a solid state, fusing the threads together to leave a minimum remaining pore fraction. The thermal conductivity of the thermal insulation will depend highly on the temperature exposure and the onset of crystallisation, sintering or melting of the insulation. Hence, the sintering effect will increase the thermal conductivity of the thermal insulation [25].

The sintering process may start at temperatures that are approximately two-thirds of the absolute melting temperature for ceramic materials [26]. Hence, porous ceramic materials may be expected to start the sintering process at temperatures well below the actual melting point.

### 2.3. Furnace Testing up to 1200 °C

To investigate the thermal insulation dimensional changes and the breakdown temperature, which is defined as a considerable change in physical dimension over a limited temperature range, it was decided that a muffle furnace be used for the heat treatment. In order to minimise any elasticity issues, the thermal insulation test specimens (50 mm cubes) were pre-cut a couple of days before the heat treatment in a muffle furnace (Laboratory Chamber Furnace, Thermconcept GmbH, Bremen, Germany). The maximum temperature of the furnace was 1300 °C, i.e., well above the highest temperature (1200 °C) of interest in the present study.

**Figure 3:**
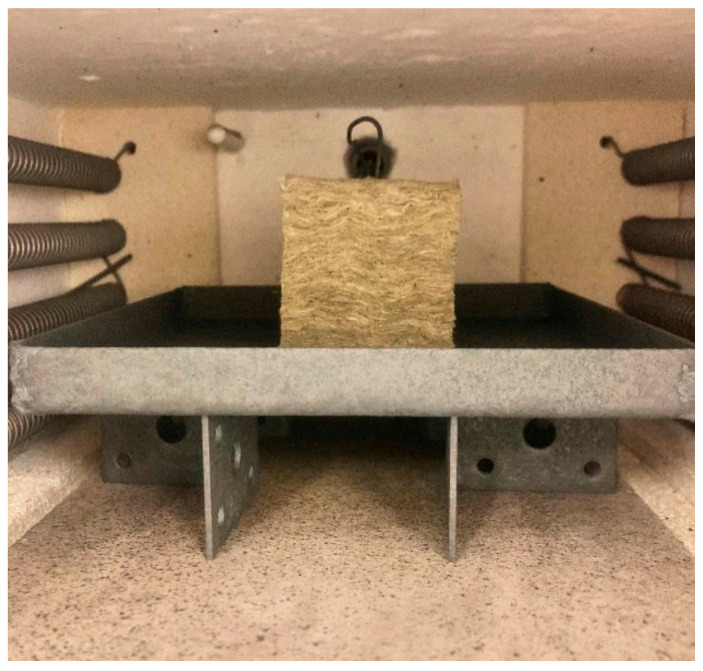
Test setup in the muffle oven.

One thermocouple (type K, mantel, 1.5 mm diameter, Pentronic AB, Västervik, Sweden) was inserted vertically into the centre of the 50 mm cubic test specimens to record the internal test specimen temperature. To record the furnace temperature, a second thermocouple was placed in the upper part of the furnace. The test specimen was placed on a steel plate and lifted approximately 35 mm above the 15 mm thick bottom plate, as shown in Figure 3, to allow for uniform heating of the specimen. In order to minimise any thermal radiation shadowing effects, only one test specimen was placed in the furnace for each heat exposure test.

The heat treatment of the test specimen was performed for temperatures in the range of 700 to 1200 °C, as presented in Table 1. The heating rate of the oven was set to 15 K/min and the test specimens were kept at the respective holding temperatures for 30 min.

After each heat treatment and cooling to below 100 °C, the test specimen was carefully removed from the furnace and the length and width were recorded at three locations at each of the four vertical faces. The average width and height were reported for each test specimen.

### 2.4. Thermogravimetric Analysis and Differential Scanning Calorimetry

To support the results from the furnace testing and to get more detailed information about the breakdown processes, samples of the thermal insulation were tested in a simultaneous TGA/DSC apparatus (Simultaneous Thermal Analyzer STA 449F3, NETZSCH, Selb, Germany). Prior to the sample preparation, a larger sample was taken from the insulation mat, crushed and mixed well into one large batch, from which each sample was taken. This was done to, as far as possible, even out minor variations in the chemical composition of the different spun layers. The sample mass was approximately 12 mg (±1 mg). The TGA/DSC tests were run at heating rates of 5, 10, 20 and 40 K/min from room temperature to 1250 °C. Three tests were run at each heating rate. The tests were conducted in a nitrogen atmosphere to prevent air oxidation.

### 2.5. Transient Plane Source Thermal Conductivity Measurements

The thermal conductivity of the virgin thermal insulation was given by the manufacturer, as shown in Table A2. Test specimens for thermal conductivity measurements were also initially 50 mm cubes and were heat-treated in a similar way as previously described. The highest heat treatment temperature for these test specimens was 1200 °C. Post heat treatment, TPS [27,28] was used to record the thermal conductivity of each test specimen at room temperature. However, no thermocouple penetrated these test specimens since that would have left a hole when removed and thus disturbed the TPS thermal conductivity measurements.

## 3. Results

### 3.1. Heat Treatment of 50 mm Thermal Insulation Cubes to 1200 °C

**Figure 4:**
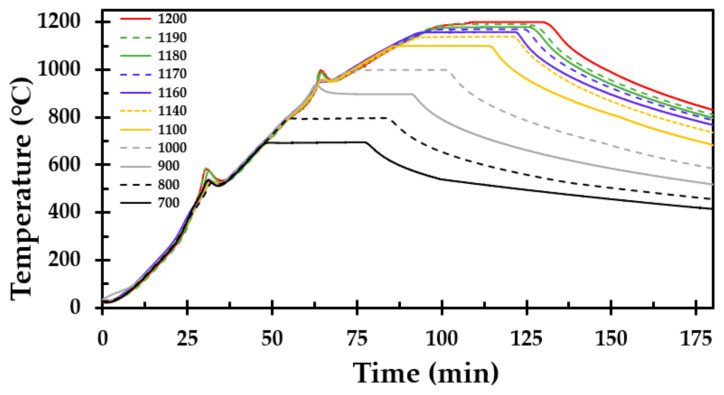
Measured temperature as a function of time, measured at the center of the test specimen, for each holding temperature presented in Table 1.

During the heat treatment in the muffle furnace, the temperature at the center of the insulation sample was recorded. The recorded temperature as a function of time for each defined holding temperature is presented in Figure 4. The temperature curves show the heating of the thermal insulation, the 30 min holding time and the subsequent cooling of the furnace. Two temperature peaks were observed in all the tests, i.e., two exothermic reactions. The peak temperature in the reaction varied, which may be explained by the variations in the amounts of dust binder and Bakelite in each test cube. The first peak started at about 300 °C, with a peak in temperature between 525 and 587 °C. The second peak started at approximately 870 °C, with a peak temperature between 930 and 990 °C. Thus, the second peak was only observed in test specimens treated at 900 °C and above. As stated in [11], the first peak (exothermic reaction) may be explained by the combustion of the dust binder due to the ambient air atmosphere in the furnace. The second exothermic peak may be explained by the crystallisation of the amorphous silica (SiO_2_) in the thermal insulation.

**Figure 5:**
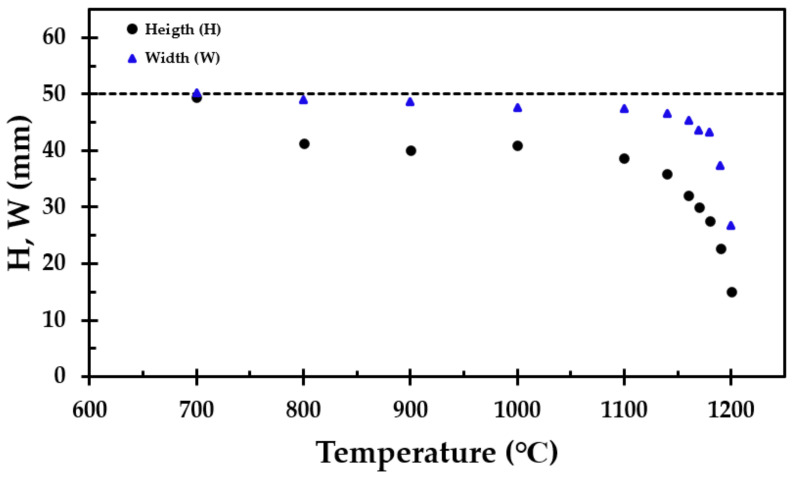
Height (H) of the test specimen (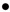) and width (W) of the test specimen (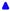) after the heat treatment. The height and the width are the average value of three measurements at each side of each of the four vertical faces.

After the heat treatment, the height of the originally 50 mm high cube of thermal insulation was recorded at three locations at each of the four vertical faces. Similarly, the width of the test specimen was recorded horizontally at three elevations for each of the four vertical faces. There was some variation in height and width in the heat-treated test specimens, hence an average value had to be used. The results from the average measurements from the height (H) and width (W) of the heat-treated thermal insulation are presented in Figure 5.

**Figure 6:**
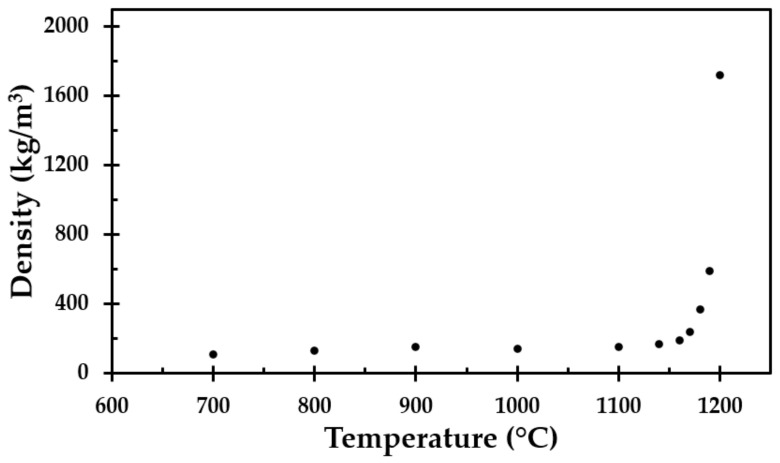
Density as a function of the heat exposure temperature.

Based on the obtained average height and width of each cube, an estimation of the post-heat-treatment volume was made. The mass of each specimen was also recorded, allowing for the density to be calculated for each cube, as presented in Figure 6.

**Figure 7:**
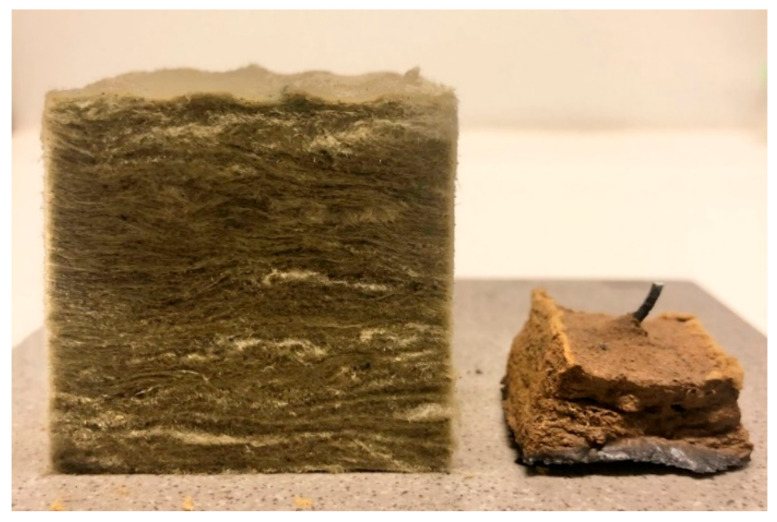
Virgin test specimen (50 mm cube) (left) and heat-treated to 1200 °C (right), including the thermocouple that had to be cut when the specimen was removed from the furnace.

A minor decrease in the test specimen height was observed for heat treatment temperatures up to 1100 °C, similar to previously published results [11], which were limited to a maximum temperature of 1100 °C. Above this temperature, the results of the present study clearly show that significant degradation of the thermal insulation started at temperatures just above 1100 °C. There also seemed to be a total breakdown at 1200 °C, as evidenced by a conspicuous increase in the post-heat-treatment density. The virgin test specimen and the test specimen heat-treated up to 1200 °C are presented in Figure 7.

**Figure 8:**
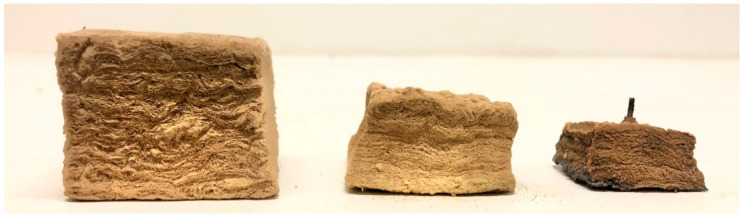
Test specimens after furnace heat treatments up to 1100 °C (left), 1190 °C (middle) and 1200 °C (right), as seen from the side.

**Figure 9:**
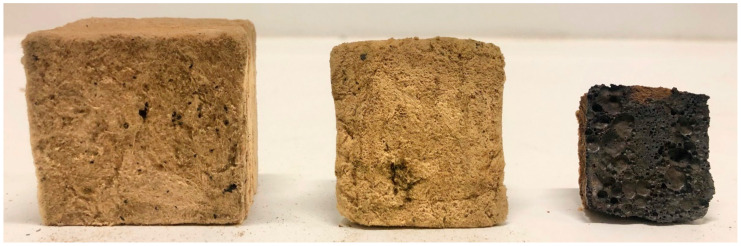
Test specimens after furnace heat treatments up to 1100 °C (left), 1190 °C (middle) and 1200 °C (right), as seen from the bottom of the insulation.

The test specimens after heat treatments up to 1100 °C, 1190 °C and 1200 °C, from left to right, respectively, are shown in Figure 8 and Figure 9. After the heat treatment at 1190 °C, the test specimen had lost 55% of its original height and 25% of its original width, while the heat treatment at 1200 °C resulted in a 76% reduced height and a 46% reduced width. When increasing the heat treatment temperature from 1190 °C to 1200 °C, the thermal insulation material post heat treatment changed in morphology from a chalky consistency to resembling a hard, but still somewhat porous, stone. This was clearly shown in the calculated density, which increased from 589 to 1721 kg/m^3^ due to the 10 °C increase in heat treatment temperature from 1190 °C to 1200 °C.

### 3.2. Thermogravimetric Analysis

**Figure 10:**
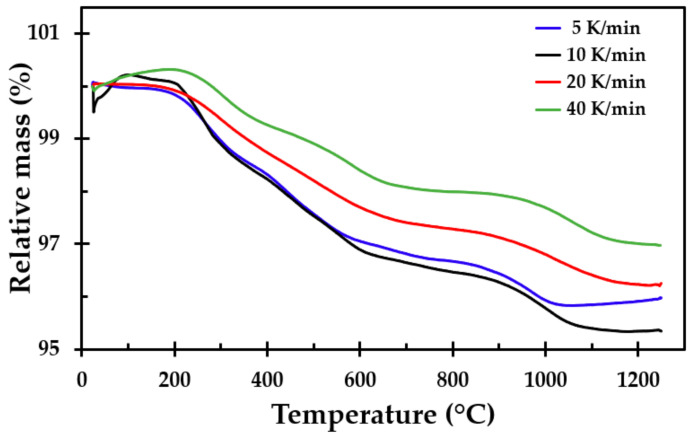
Thermogravimetric analysis of the thermal insulation in a nitrogen atmosphere.

**Figure 11:**
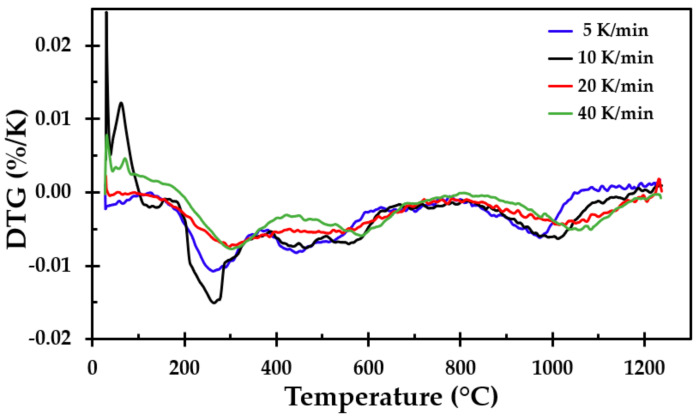
Differential thermogravimetric (DTG) analysis of the results presented in Figure 10.

Thermogravimetric analysis was conducted from ambient temperature up to 1250 °C. The heating rates were 5, 10, 20 and 40 K/min. The samples for the TGA testing were made from the same insulation mat as the muffle furnace testing. The approximate mass loss was between 3 and 4.3%, as shown in Figure 10. The differential thermogravimetric (DTG) analysis, which is the derivative of the TGA curve, is presented in Figure 11.

The mass loss of the insulation started at approximately 180 °C, with a local minimum value between 260 and 290 °C. This may be explained by the release of the dust binder. The mass losses at higher temperatures were most likely due to the Bakelite binder and possibly some released chemically bound water, with the most conspicuous peak observed at about 1000 °C.

### 3.3. Differential Scanning Calorimetry

**Figure 12:**
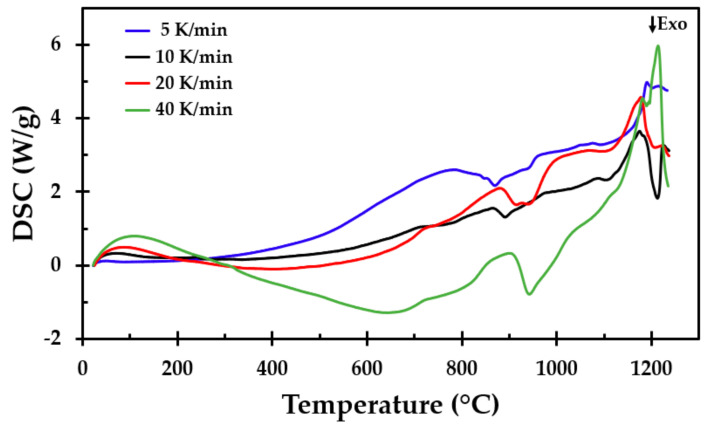
The differential scanning calorimetry (DSC) results as a function of temperature.

Simultaneously with the TGA measurements, DSC analyses were performed from ambient temperature up to 1250 °C at heating rates of 5, 10, 20 and 40 K/min. The results from the DSC analysis are presented in Figure 12. An exothermic reaction started between 800 and 900 °C. An endothermic peak was observed at, or just above, 900 °C. A very conspicuous endothermic reaction was observed starting at approximately 1120 °C, with a maximum local peak between 1170 and 1206 °C.

The minimum and maximum values from the exothermic and endothermic reactions for the three conducted tests at each heating rate are presented in Table 2, in addition to the heat flow values at the endothermic peaks. There were some variations in the peak value, depending on the heating rate, but there was no clear trend associated with the heating rate and peak temperature.

### 3.4. Thermal Conductivity Measurements

**Figure 13:**
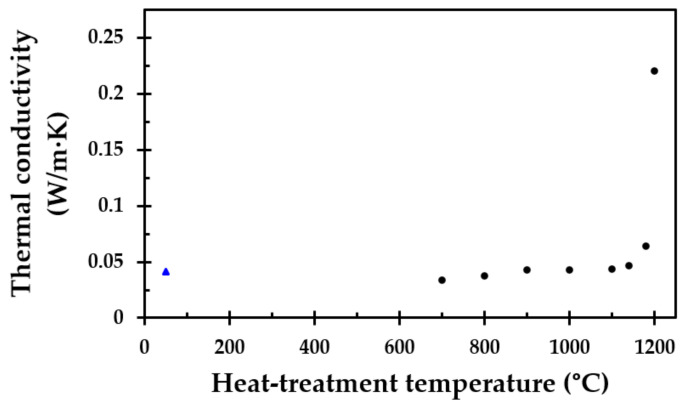
Recorded room temperature thermal conductivity as a function of the test specimen heat treatment temperature. The thermal conductivity at 50 °C (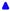) was from Appendix A, Table A2.

TPS [27,28] was used to record the room temperature thermal conductivity of the heat-treated test specimens. The obtained results as a function of the heat treatment temperature are shown in Figure 13. The thermal conductivity increased with the heat treatment temperature in a similar manner to the recorded density, as presented in Figure 6, i.e., it increased greatly when heat-treated to temperatures above 1150 °C. This was most likely due to the increasing level of sintering and partly due to melting, as evidenced by the endothermic peak in Figure 12 at these high temperatures. The most conspicuous change was observed when the heat treatment temperature was 1200 °C, i.e., where more melting occurred during the heat treatment.

## 4. Discussion

Previous studies have shown that the type of thermal insulation tested in this study survived well when heated in a muffle oven to temperatures up to 1100 °C [11]. The objective of the present study was to investigate the performance of the thermal insulation when exposed to temperatures up to 1200 °C, i.e., temperatures associated with fire heat fluxes of about 350 kW/m^2^. The focus was on finding the breakdown temperature of the thermal insulation. Small scale jet fire testing has proven that the thermal insulation alone may serve as passive fire protection of a 16 mm steel wall [10,11]. The previous jet fire tests showed a complete breakdown of the insulation at the most exposed locations. To determine the breakdown temperature and explain the observations of the insulation after the small-scale jet fire testing, muffle furnace tests up to 1200 °C were performed, as well as TGA/DSC to 1250 °C in a nitrogen atmosphere.

The results from the furnace testing showed the same trend as in [11] up to 1100 °C, which was the upper-temperature limit of that study due to furnace limitations. However, at heat treatment temperatures above 1100 °C, the height of the originally 50 mm cubes started to shrink significantly with increasing heat treatment temperature. From 1160 °C, the width of the test specimens also decreased considerably. The thermal insulation fibers gradually sintered/melted more and more together, and the insulation transformed from being a porous material to a hard, stony consistency when heat-treated to 1200 °C. This was also reflected in the calculated density of the thermal insulation cubes post heat treatment. The density close to tripled due to the heat treatment at 1200 °C compared to 1190 °C. It was clearly shown from the results that heating to 1200 °C is very close to, or even at, the melting point, or eutectic temperature, of the insulation.

Mixtures of inorganic salts, such as the investigated thermal insulation, will not show a defined melting point, but rather an extremely complex phase diagram with several eutectic points. It is therefore expected to gradually melt, without a defined melting temperature. Heat treatment of the test specimen cubes to temperatures above 1200 °C might have resulted in a glass-like substance. This was, however, outside the scope of the present study but may be interesting for future studies.

The thermal conductivity of the virgin thermal insulation was clearly dominated by heat radiation through the pores at moderately elevated temperatures. At higher temperatures, the onset of sintering increased the solid–solid contact phase, improving the true thermal conductivity of the material. This was confirmed by the room temperature thermal conductivity obtained in the present study. However, at a still higher pore fraction, it would be expected that at elevated temperatures, the pore radiation would continue to dominate the effective thermal conductivity. When heat-treated to 1200 °C, the significant increase in density indicated that the pore fraction must be very low. In this stage, the thermal conductivity may not be very dependent on the pore radiation, i.e., not show a very strong dependency on the absolute temperature to the third power. To validate this assumption, the thermal conductivity of the heat-treated test specimens must be recorded at elevated temperatures. This was, however, outside the scope of the present study.

Heat treatment up to 1100 °C revealed some loss in height, approximately 22%. However, little change in the width of the material was observed up to this temperature, i.e., it was not expected that the insulation mat will crack open at temperatures below 1100 °C. When heating to 1180 °C, the loss in width was still below 14%. However, when heating up to 1190 °C, there was a significant loss in width, i.e., 25%.

Due to the heat treatment at 1200 °C, the insulation cube lost 76% of its height and 46% of its original width, explaining the observed cracks and openings in the insulation mat after the small scale jet fire testing presented in [10,11]. In addition to the shrinkage in height (thickness) and the increase in thermal conductivity due to the sintering, in a severe fire scenario, there will be radiant heat transfer through the cracks and openings. Hence, with more and wider cracks, more radiant heat may bypass the thermal insulation, leading to excessive heating of any fire-exposed objects.

In the furnace heat treatment tests, two exothermic peaks were observed. The first of these may be explained by the combustion of the dust binder material at about 300 °C. There were some variations in the peak temperature of the reaction, which may be explained by differences in the chemical compositions between the samples. This exothermic reaction at approximately 300 °C, observed during heat treatment, was not present in the DSC tests. This may be explained by the air access and combustion in the furnace and the inert gas (nitrogen) atmosphere during the DSC tests. In an oxygen atmosphere, both the first and the second peaks were observed [11]. The second peak at about 900 °C may have been due to the Bakelite combustion or a recrystallisation process of the involved inorganic salts.

The TGA showed that only small amounts of the material vaporised during heating, i.e., approximately 4% of the mass was lost. This was also seen when observing the density of the heat-treated test specimens, where there was little change in the mass of the test samples due to the heat treatment, i.e., the density increased as the insulation sintered and finally started to partly melt. The differences in mass loss recorded using the TGA for the different heating rates were also observed in repeated tests at each heating rate. In their study of different stone wool insulations, Livkiss et al. [16] also observed similar discrepancies. They explained these differences using the inhomogeneity of these types of material. We also agree with this assumption since TGA and DSC testing is constrained to test samples that are a few milligrams in size.

**Figure 14:**
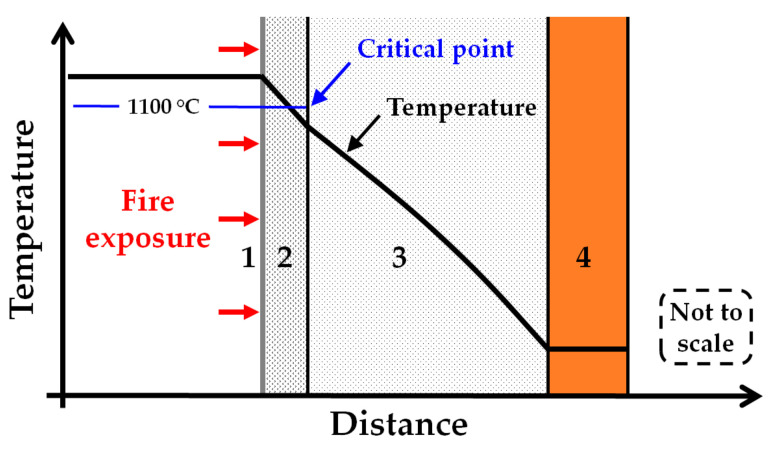
Sketch of fire exposure, weather protection cladding (1), the layer of heat-resistant insulation (2), thermal insulation (3) and the object to be protected from fire exposure (4). The critical point to be kept below is a temperature of 1100 °C, which is marked on the figure.

The heat treatment in the present study involved a holding time of 30 min at all heat treatment temperatures. It is interesting to notice that if the temperature of the thermal insulation was kept at, or below, 1100 °C, it could stay quite intact for at least the 30 min heat exposure. It started to significantly break down only at temperatures above 1100 °C. Hence, if arranged in a passive fire protection system such that it will not exceed 1100 °C, it may contribute significantly as passive fire protection in addition to its intended function as thermal insulation. A sketch of such an arrangement is presented in Figure 14. The critical point to be kept below is 1100 °C, which is marked on the figure. Unless the object to be protected is internally cooled by, e.g., depressurisation, the temperatures of the system will gradually increase, but these layers of protection may be designed to offer the required protective capacity for the desired time.

It should be noted that different batches of industrial thermal insulation may show slightly different high-temperature performances. Thermal insulation that varies significantly from the chemical composition presented in the present study may show very different high-temperature properties. Care should therefore be taken before using such materials for fire protection. The 50 mm cubes that were heat-treated in the muffle furnace and the TGA/DSC analysis both provided results supporting the conclusions in the present study. When considering industrial thermal insulation for that also supplies some passive protection in fire situations, it may in the future be sufficient to use only one of these methods for a preliminary evaluation of the potential passive fire protection capability.

In the future, it would be beneficial to do further fire testing of, e.g., small-scale jet fire tests [10,11] with an additional protective layer, as demonstrated in Figure 14. When properly chosen, this layer may then keep the exposed thermal insulation below the breakdown temperatures, thereby ensuring that it may contribute towards providing significantly prolonged fire protection.

Heat treatment tests in a muffle furnace that test other types of insulation, e.g., mineral-based passive fire protection or different types of Rockwool insulation, could give more information about future possibilities. It would also be beneficial to measure the thermal conductivity of the thermal insulation at elevated temperatures. This could give the information required for developing a numerical model of the thermal insulation performance when exposed to fires, with or without a protective layer, as indicated in Figure 14.

## 5. Conclusions

Except for a 20% vertical (*z*-direction) shrinkage at 800 °C, the present study showed that the properties of the analysed thermal insulation did not change much when heat-treated to 1100 °C, which is associated with pool fire heat flux levels (250 kW/m^2^). However, when heat treated to 1200 °C, which is associated with jet fire heat flux levels (350 kW/m^2^), the thermal insulation changed greatly. The density and room temperature thermal conductivity increased from 140 to 1700 kg/m^3^ and from 0.041 W/m∙K to 0.22 W/m∙K, respectively. The horizontal (*x*- and *y*-direction) shrinkage that took place at heat treatment temperatures above 1180 °C created gaps in the insulation, i.e., allowed for unrestricted radiant heat flow at exposed locations. However, if a thin layer of thermal insulation that is robust to temperatures above 1200 °C is placed at the heat exposed side, the studied thermal insulation may provide significant passive fire protection. It is recommended that this is tested in future studies.

## Figures and Tables

**Table 1:** Number of tests at each holding temperature.

Holding Temperature (°C)	Number of Tests
700	2
800	2
900	2
1000	2
1100	2
1120	1
1140	2
1160	1
1170	1
1180	2
1190	1
1200	2

**Table 2:** The recorded temperatures at the exothermic (T_l,exo_) and endothermic (T_p,endo_) DSC peaks of Figure 12 and the recorded heat flows at the peaks for each run.

Run	Heating Rate (K/min)	T_l,exo_ (°C)	T_p,endo_ (°C)	DSC (W/g)
1	5	848.0	1210.5	3.18
2	5	873.9	1190.9	4.98
3	5	922.9	1209.9	6.48
Average		881.6	1203.8	4.88
1	10	889.6	1202.6	1.82
2	10	893.9	1174.9	3.65
3	10	917.9	1175.9	4.73
Average		900.4	1184.4	3.40
1	20	920.2	1156.2	1.85
2	20	943.2	1177.2	4.56
3	20	950.0	1175.0	5.59
Average		937.8	1169.4	4.00
1	40	935.1	1216.3	4.47
2	40	942.7	1213.7	5.97
3	40	938.3	1190.3	6.02
Average		938.7	1206.7	5.49

## Appendix A

The technical data for the thermal insulation is given in Table A1 and Table A2. The chemical composition of the thermal insulation is given in Table A3.

**Table A1:** Technical data for the Rockwool pipe section mat thermal insulation [29].

Name	Description	
Material	Stone Wool	
Operating Range	−40 to 700 °C	
Name	Performance	Norms
Maximum Service Temperature	700 °C	EN 14706
Reaction to Fire	Euroclass A1	EN 13501-1
Nominal Density	140 kg/m^3^	EN 1602
Water Absorption	≤1 kg/m^2^ ≤20 kg/m^3^	EN 1609
		BP 172
Water Vapor Diffusion Resistance	Sd > 200 m	EN 12086
Air Flow Resistivity	>60 kPa∙s/m^2^	
Designation Code	MW EN 14303-T4-ST(+)700-WS1-MV2	EN 14303

**Table A2:** Thermal conductivity of the thermal insulation studied (Rockwool ProRox PSM 971, 50 mm) [29].

Temperature (°C)	Thermal Conductivity (W/m·K)
50	0.041
100	0.046
150	0.054
200	0.064
250	0.075
300	0.088
350	0.106

**Table A3:** Data for the thermal insulation studied (Rockwool ProRox PSM 971. 50 mm) [30].

Name	Product	Percentage
Dust Binder ^1^	Oil product	<0.5%
Binder ^1^	(C_6_H_6_O·CH_2_O)_N_	2.5% (±0.4%)
Bulk Oxide	SiO_2_	40.6–44.6%
Bulk Oxide	Al_2_O_3_	17.4–20.4%
Bulk Oxide	MgO + CaO	23.9–27.9%
Bulk Oxide	Fe_2_O_3_	5.5–8.5%
Bulk Oxide	Na_2_O + K_2_O	1.3–4.3%
Bulk Oxide	TiO_2_	0.6–2.6%
Bulk Oxide	P_2_O_5_	Max. 1.2%

^1^ The binder calorific value is 27 MJ/kg according to ISO 1716.
